# Supplementary material for: A lightweight durable full-body electrical stimulation suit for haptic feedback and therapeutic applications
Source: Nat Commun. 2026 Apr 2;17:4764. doi: 10.1038/s41467-026-71171-y (PMC13216600; doi:10.1038/s41467-026-71171-y)
Supplement: Supplementary file 1 — Supplementary Information [file 41467_2026_71171_MOESM1_ESM.pdf]

Supplementary Information for:

A Lightweight Durable Full-Body Electrical Stimulation Suit for Haptic Feedback and Therapeutic Applications

Jin Hee Hwang[1†], Sun Hong Kim[2†], Ju-Hwan Kim[3,4†], Jae-young Yoo[5†], Jungmin Seo[6], Geonoh Choe[1], Jae Min Lee[1], Byungkeun Choi[1], Sungjun Park[7], Joohoon Kang[8], Sang Min Won[9], Jeonghee Kim[1,6,*], Dong-Wook Park[3,4,*] and Yei Hwan Jung[1,6,10,*]

[1]*Department of Electronic Engineering, Hanyang University, Seoul 04763, Republic of Korea*

[2]*Department of Chemical Engineering, University of Seoul, Seoul 02504, Republic of Korea*

[3]*School of Electrical and Computer Engineering, University of Seoul, Seoul 02504, Republic of Korea*

[4]*Center for Semiconductor Research, University of Seoul, Seoul 02504, Republic of Korea*

[5]*Department of Semiconductor Convergence Engineering, Sungkyunkwan University, Suwon, Republic of Korea*

[6]*Department of Artificial Intelligence Semiconductor Engineering, Hanyang University, Seoul 04763, Republic of Korea*

[7]*Department of Electrical and Computer Engineering, Ajou University, Suwon 16499, Republic of Korea*

[8]*Department of Chemical and Biomolecular Engineering, Yonsei University, Seoul 03722, Republic of Korea*

[9]*Department of Electrical and Computer Engineering, Sungkyunkwan University, Suwon 16419, Republic of Korea*

[10]*Institute of Nano Science and Technology, Hanyang University, Seoul 04763, Republic of Korea*

[†]*These authors contributed equally to this work*

*To whom correspondence should be addressed. E-mail: jkim448@hanyang.ac.kr, dwpark31@uos.ac.kr, yjung@hanyang.ac.kr

Supplementary Notes

Supplementary Note 1. Printing accuracy on textile of Ag-PU conductor

To assess printing accuracy on the textile substrate, Ag-PU conductors (30 mm trace length) were screen-printed using stencil masks with nominal line widths of 100, 250, and 500 μm (Supplementary Fig. 3a,b). All traces remained electrically conductive, and resistance decreased with increasing line width, confirming reliable interconnection and defining the achievable printing fidelity for textile-based conductive arrays. While prior studies report improved pattern fidelity using approaches such as rheology-engineered inks, in-textile photolithography, or thermal transfer printing[1–3], these methods typically require additional materials, specialized equipment, and/or extra processing steps. Screen printing was therefore selected as a practical compromise between scalability, throughput, and cost for large-area textile fabrication, while remaining compatible with commercial fabrics.

Supplementary Note 2. Electrical properties of printed Ag-PU conductors

Supplementary Figure 5 shows the absolute resistance of the printed Ag-PU conductors as a function of pattern geometry. For the 2 mm trace width used throughout this study, a 30 mm-long conductor exhibits an average resistance of ~1.9 Ω. We also benchmarked the conductivity of our Ag-PU conductors against previously reported Ag-based inks for textile electronics (Supplementary Table 1) confirming that our conductivity is comparable to representative textile-based Ag inks reported in prior work. Beyond conductivity, our ink enables hydrogen-bond–mediated adhesion to hydrophilic hydrogels without the additional surface treatments often required for hydrophobic Ag composites[4–6], and it can be fabricated without high-temperature post-processing, supporting scalable integration in textile-based wearable systems. To evaluate durability, 13 Ag-PU conductor patterns (30 mm × 2 mm) were fabricated on the textile and subjected to a washing test consisting of four 10-min cycles under magnetic stirring in water at 650 rpm (Supplementary Fig. 6).

Supplementary Note 3. The optimization of the DMCH electrode thickness

The DMCH electrode thickness was optimized by balancing mechanical integrity and electrical stability under compression. As shown in Supplementary Figure 11, the 1 mm DMCH could not sustain stable measurements above 1.3 N, whereas the 2 mm DMCH remained mechanically intact and electrically stable up to 5 N, exhibiting the smallest resistance change. Consistently, Supplementary Figure 12 shows that the 1 mm DMCH has lower conductivity ($\sigma \approx 0.025\ mS\cdot cm^{-1}$) than the thickness-insensitive regime observed for thicker DMCH ($\sigma \approx 0.12$–$0.14\ mS\cdot cm^{-1}$), consistent with prior reports on PEDOT:PSS-based electrodes[7–12]. Together, these results indicate that 2 mm is the minimum thickness required to ensure a robust conductive path and reliable electrical performance under compressive loading up to 5 N.

Supplementary Note 4. Water vapor transmission rate test

Water vapor transmission was measured using a 10 ml beaker (radius = 43 mm) filled with deionized (DI) water. Tests were conducted under controlled environmental conditions—37 °C and 19 % relative humidity—for 24 h using a temperature- and humidity-regulated oven (OF-02PW, JEIO TECH). Each sample, standardized to 2 cm × 2 cm in size, was affixed over the beaker opening and sealed with rubber bands. The cumulative weight loss was recorded using a high-precision analytical balance (WBA-220, DAIHAN Scientific). WVTR was calculated using the following equation:

$$\mathrm{WVTR} = \frac{\Delta W(\mathrm{g})}{At(\mathrm{m^2\ h})} \tag{1}$$

where ΔW is the weight loss (g), A is the sample area (m^2), and t is the duration (24 h).

Supplementary Note 5. Sweat user test

Four participants (both male and female) in their 20s, with a mean age of 24.75 years (two females; Supplementary Fig. 36d), took part in a user test designed to evaluate the effects of perspiration on electrical stimulation. The objective was to assess the amplification of current signals caused by sweat and the accompanying spread of stinging sensations across the skin. Prior to the exercise, participants washed their forearms and attached electrodes in a dry state. A standardized stimulation protocol of 10 V at 5 Hz—common across all participants—was applied both before and after physical activity to measure current changes. Participants then engaged in a 10-min run under controlled environmental conditions (22 °C, 72 % relative humidity). To assess breathability, participants rested in a seated position for 10 min at 22.3 °C and 55 % humidity, followed by another current measurement. The total stimulation duration did not exceed 10 min. Immediately afterward, participants evaluated the level of itchiness induced by sweat accumulation. This assessment was subjective and based on relative perception. Stimulation was applied sequentially using the textile-based haptic electrode, TENS electrode-1, and TENS electrode-2, and all evaluations were conducted collectively after the test.

Supplementary Note 6. Peeling force measurement

To assess adhesion strength, Ag-PU conductor samples were fabricated by partially overlapping the DMCH hydrogel onto textiles fully printed with Ag-PU ink. For the Ecoflex-Ag comparison samples, Ecoflex (Ecoflex 00-30, Smooth-On) was first mixed with an organic solvent (MIBK, 2-methyl-4-pentanone, Sigma-Aldrich) for 30 min. Ag flakes (~1.3 μm, DSF-500 MWZ-S, Daejoo Electronic Materials Co., Ltd.) were then added and mixed via mechanical stirring for 2 h. Once the conductive inks were printed onto the textiles, DMCH was partially laminated on top. All samples were cured in an oven at 60 °C for 10 min. The adhesion strength between the textile and DMCH was evaluated using a universal testing machine (Instron-5543, INSTRON) by stretching the samples until delamination occurred.

Supplementary Note 7. Components of the control circuit

The entire control circuit was assembled on a custom-designed, double-sided flexible printed circuit board (FPCB) manufactured by PCBway. The board featured an 18-μm thick metal pattern with an immersion gold surface finish. Component soldering was carried out using a soldering iron (WE1010NA, Weller) and a hot air rework station (EX-930, Exso). Solder wire (91-6040-9013, Kester) and solder paste (TS391LT, Chip Quik) were used during the bonding process. The microcontroller unit (MCU) (ISP1807, Insight SIP), equipped with an integrated BLE antenna, provided compact wireless communication capabilities. A quad precision operational amplifier (OPA4191, Texas Instruments), a digital potentiometer (MAX5419, Analog Devices), and various resistors and capacitors were firmly mounted on the board to support electrical stimulation signal generation.

Power for the amplification block was supplied by a DC-DC switching converter (MCP1663, Microchip Technology) in combination with an inductor (LPS6235-123MRC, Coilcraft), a diode (UPS5819, Microchip Technology), and other passive components. The power management circuit included a low-dropout (LDO) linear regulator (NCV8537MN180R2G, Onsemi), a linear charger for a lithium-polymer battery (LTC4065LEDC, Analog Devices), and a buck converter (LM5166XDRCR, Texas Instruments) for wireless DC-to-DC conversion. Additional components included a rectifier diode (BAS4002ARPPE6) and an inductor (LPS5030-562MRB, Coilcraft), along with a silicone-coated wire-wound loop coil for inductive energy harvesting.

Supplementary Note 8. Control circuit operation

The haptic system features wireless communication and is operated through a graphical user interface (GUI), allowing users to control and deliver electrical stimulation feedback seamlessly. The custom-designed electrical stimulation circuit supports independent pulse waveform generation of up to 30 V across a 16-channel electrical stimulation array, with four analog-to-digital converter (ADC) ports assigned for pressure sensing (Supplementary Fig. 30). The system is powered by a rechargeable lithium-polymer (Li-Po) battery, regulated to a stable 3.3 V using a LDO linear regulator. A MCU with an integrated 2.4 GHz antenna enables BLE communication, eliminating the need for external antennas and conserving space. Each general-purpose input/output (GPIO) pin of the MCU produces modulated square waves, which are fed into an amplification circuit. The circuit is based on a non-inverting amplifier topology, equipped with a digitally programmable potentiometer connected via an I^2C interface. This configuration allows real-time tuning of the amplifier gain per channel, enabling precise output control up to 30 V. The digital potentiometer adjusts the resistance at the negative input of the operational amplifier by communicating with the MCU, achieving the desired gain dynamically. The amplifier, featuring a rail-to-rail output, operates with a boosted supply voltage from a DC converter, which elevates the LDO-regulated 3.3 V to the required 30 V stimulation voltage, ensuring consistent output in response to control signals (Supplementary Fig. 31). To support real-time impedance compensation, the MCU's integrated 14-bit ADC is coupled with a pressure sensor, enabling feedback control. The system's flexible printed circuit board (FPCB), made of polyimide, is divided into four segments interconnected by stretchable serpentine traces, enhancing mechanical flexibility and wearability (Supplementary Fig. 32a–c). Further segmentation was avoided to maintain an optimal balance between board size and mechanical stretchability. Power management is handled by an integrated circuit coupled with a receiver-loop antenna, enabling continuous operation. Energy harvesting is achieved via an

inductively coupled coil adjacent to the board. When placed near a near-field communication (NFC) power transmission antenna, the coil captures energy to recharge the Li-Po battery, supporting wireless recharging capabilities (Supplementary Fig. 32d).

Supplementary Note 9. Tremor treatment

Hand tremors were accurately captured using the BNO055 IMU. The controllers embedded with this sensor acted as BLE peripherals, transmitting 3-axis accelerometer data, 3-axis orientation data, and switch status to an NRF52832 receiver. These data enabled quantitative and objective evaluation of tremor severity. The receiver relayed the wireless data to a Unity platform using the UART protocol. To assess tremor characteristics, the 3-axis accelerometer data were processed by computing the root mean square (RMS) of the combined signals. A 4th-order Butterworth filter with a bandpass range of 4–12 Hz was applied to isolate frequency components relevant to ET. Data were logged as time-series sequences with a sampling rate of 50 Hz (timestamps recorded every 20 ms), and included three key components: accelerometer and orientation data, movement trajectory within the VR environment, and task identification. Filtered time-domain signals were transformed into the frequency domain using a fast Fourier transform (FFT). After discarding negative frequency components, the PSD was calculated by doubling the positive frequency values to preserve total signal energy. The peak frequency in the PSD was identified as the dominant tremor frequency, while overall tremor power was quantified by integrating the PSD values within the 4–12 Hz range and normalizing the results to each participant's baseline, providing a standardized metric for pre- and post-exercise tremor evaluation.

Supplementary Note 10. Implementation of feedback

Supplementary Figure 30 shows the operation of the proposed battery-powered haptic feedback system, which features a customization framework. This system dynamically adjusts stimulation parameters based on user-specific skin impedance and applied garment pressure, effectively compensating for interindividual variability and enhancing safety and comfort. For demonstration purposes, an electrode-array-based electrotactile device was mounted on the arm (Supplementary Fig. 33a,b). Each stimulator is paired with a pressure sensor placed adjacent to the stimulation site to monitor the local garment pressure. To assess whether a single-point pressure reading can represent the garment pressure around a given arm cross-section, we conducted measurements along the arm at four cross-sectional positions (from the wrist with the smallest circumference to the upper arm with the largest circumference). At each position, garment pressure was recorded at four circumferential locations (anterior, medial/inner, posterior, and lateral/outer). Across all positions, the within-cross-section variability was small; the largest deviation was observed at the wrist, yet the variability remained within the sensor tolerance (standard deviation = 0.028 at position 1; Supplementary Fig. 34a,b). These differences are not expected to produce a meaningful change in perceived stimulation intensity, supporting the use of a single-point pressure measurement as a representative value for garment pressure within a cross-section[13,14]. A robust skin–electrode interface reduces contact impedance, allowing more consistent and effective tactile feedback. Results indicated that as pressure at the interface increased, the current flow through the skin also increased (Supplementary Fig. 26 and Supplementary Table 3b). However, threshold current requirements decreased progressively as the stimulation site moved proximally from the wrist to the shoulder (Supplementary Fig. 29a), likely due to regional differences in skin impedance and sensitivity.

To address these variations in haptic perception, a real-time voltage adjustment procedure was implemented using an integrated pressure sensor (Fig. 5f). This feedback mechanism continuously monitors the skin–electrode interface, recognizing pressure as a dominant factor influencing electrode adhesion and electrical performance. The pressure sensor converts contact impedance into measurable pressure data. The analog-to-digital converter (ADC) pins on the microcontroller unit (MCU) detect voltage changes across the variable resistor in the sensor, which are interpreted in real time as pressure values to inform dynamic voltage control. After establishing a fitting equation for the threshold current at each premeasured location, we derived the correlation between current and voltage for each participant (Supplementary Fig. 29b and Supplementary Table 3a). This relationship enabled the calculation of the required threshold current, which was then converted into the corresponding stimulation voltage. Frequency-dependent variations in average threshold current were also incorporated into the equation (Fig. 4d).

Supplementary Note 11. VR-based tremor therapy approaches

In the tomato-moving task, participants used a spoon-shaped controller to transfer tomatoes from one bowl to another. Each bowl had a diameter of 20 cm, with a center-to-center spacing of 30 cm. Using their dominant hand, participants were instructed to complete the task as quickly and accurately as possible, transferring all 10 tomatoes successfully. The spiral-drawing task[15], designed to assess fine motor control and tremor severity, required participants to draw a spiral from the center outward using a marker-type controller. This task involves continuous adjustments in orientation to navigate changing path widths, curves, and turns—making it significantly more challenging than linear or simply curved tasks. Participants were instructed to complete the task with their dominant hand, prioritizing both speed and accuracy. To reduce potential order effects, task measurements were conducted in a randomized sequence before and after electrical stimulation (Supplementary Fig. 35). Additionally, participants were not informed about the purpose or expected effects of the electrical stimulation on tremors, thereby eliminating performance bias due to anticipation.

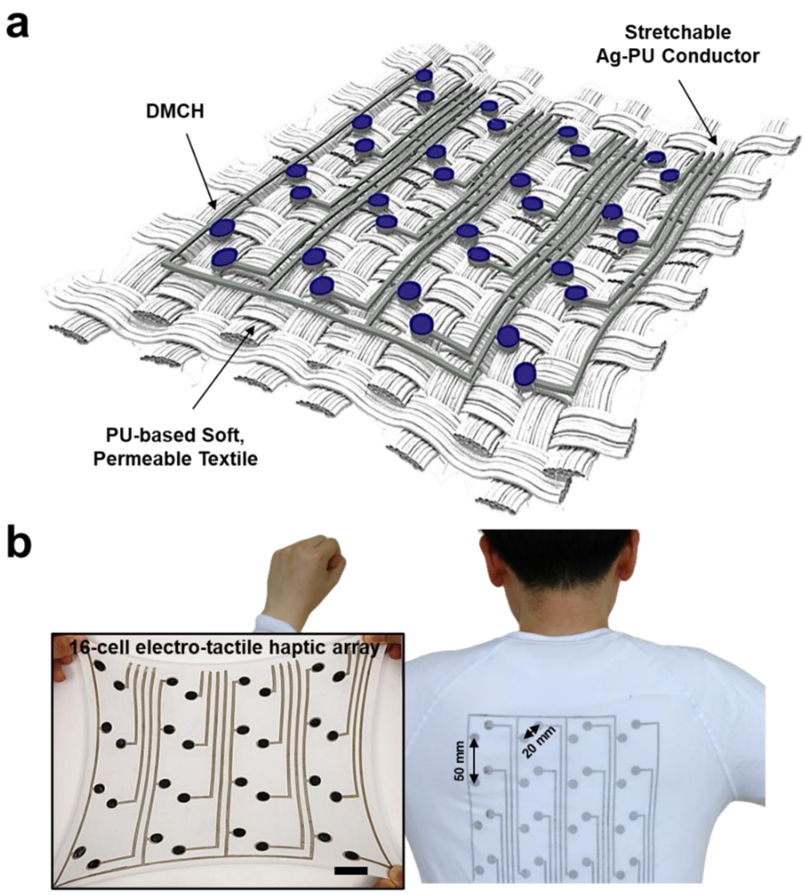

Supplementary Fig. 1 | Schematic diagram of 4 × 4 array of TESS

a, Schematic of a 4 × 4 array of TESS. A stretchable Ag-PU conductor was printed on a breathable, soft PU-based textile, and a highly conductive electrode in DMCH was securely attached on top by a strong bond. **b**, Photograph of electrical stimulation 4 × 4 array system printed on PU-based textile. The participant wearing the textile-based electrical stimulation feedback suit was fabricated on a commercial garment; the inset shows a 4 × 4 array in its stretched state. Scale bar, 20 mm.

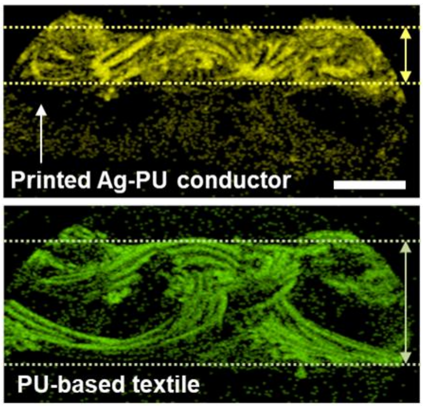

Supplementary Fig. 2 | EDS maps of PU-based textile printed with Ag-PU conductor

EDS maps of the cross-sectional area of the distribution of the Ag-PU conductor (top) and PU-based textile (bottom). In the EDS mapping, the yellow signal corresponds to the Ag L-series, and the green signal represents the O K-series. The Ag-PU conductor infiltrated the PU-based textile, resulting in a relatively dense and uniformly distributed top layer. Scale bar, 200 μm.

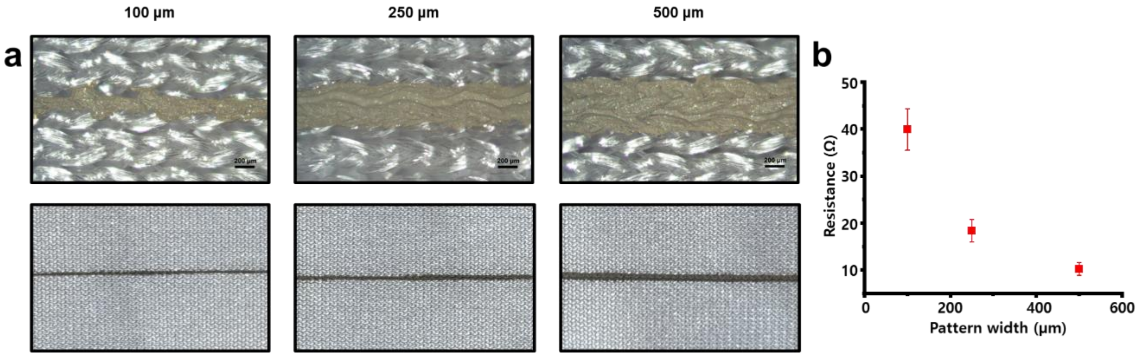

Supplementary Fig. 3 | Printing accuracy evaluation via resistance comparison across pattern widths

a, Optical microscopy images of printed widths of 108–233 μm (100 μm mask), 250–370 μm (250 μm mask), and ~500 μm with relatively uniform edges (500 μm mask). **b**, The resistance of 30 mm-long printed traces measured as a function of nominal pattern width (100, 250, and 500 μm).

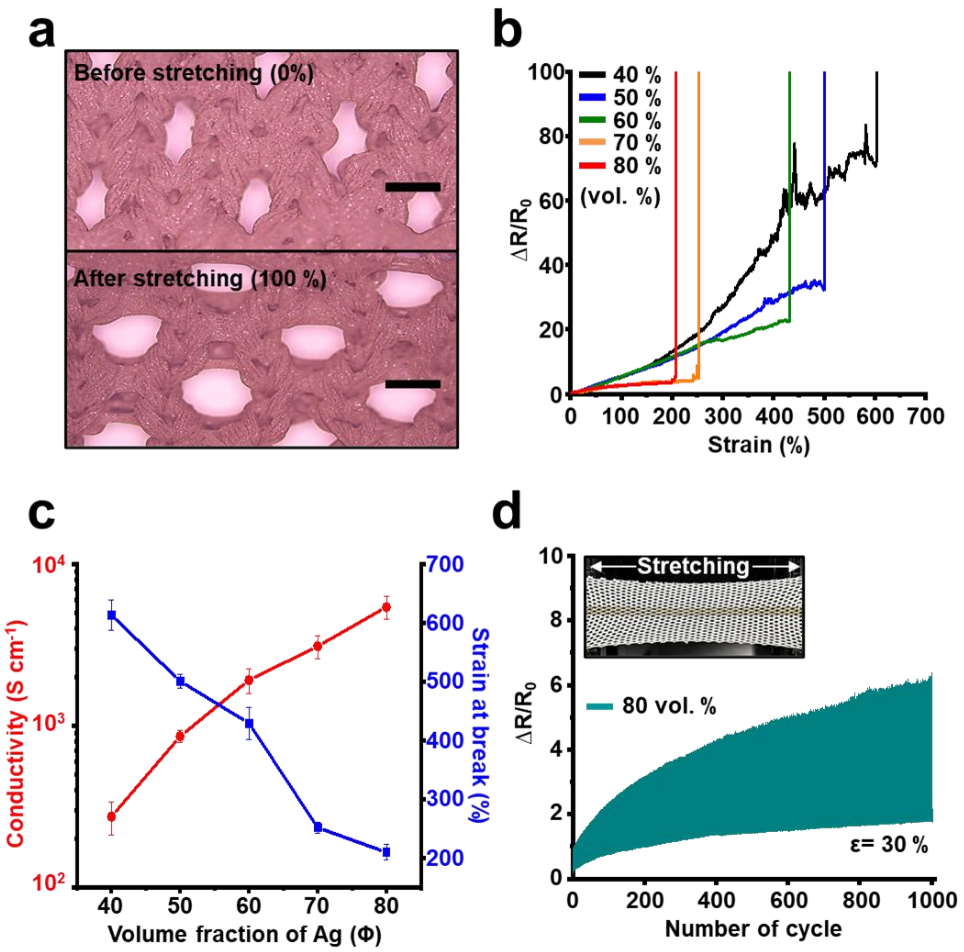

Supplementary Fig. 4 | Characterization of the conductors printed on the PU-based textile. **a**, SEM images of PU-based textile screen-printed with normalized Ag-PU conductor before (top) and after (bottom) stretching to 100% strain. Scale bar, 100 μm. **b**, Variations in resistances of strain by the volume fraction of Ag (40-80 %) in conductors. **c**, Conductivity and strain at break of composite as functions of volume fraction of Ag. **d**, Variation in the normalized resistance of the Ag-PU conductor on PU-based textile during cyclic testing at a strain of 30 %.

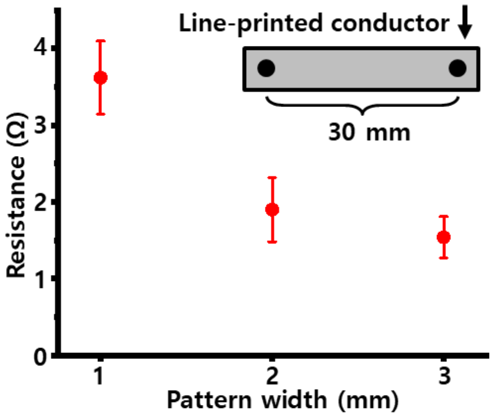

Supplementary Fig. 5 | Absolute resistance of Ag-PU conductors with varying printed patterns

Absolute resistance of 30 mm-long Ag-PU conductors as a function of printed pattern width (1, 2, and 3 mm).

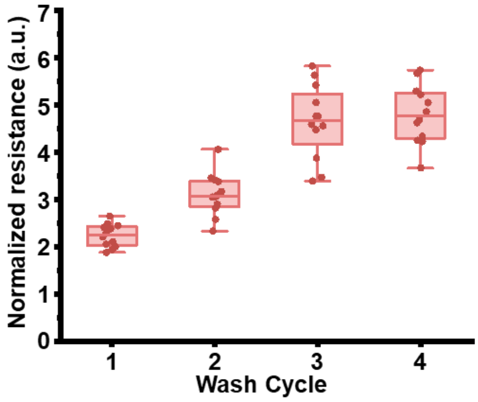

Supplementary Fig. 6 | Washability of screen-printed Ag-PU textile conductors

Change in resistance of Ag-PU conductors (30 mm length × 2 mm width) on textile after repeated washing.

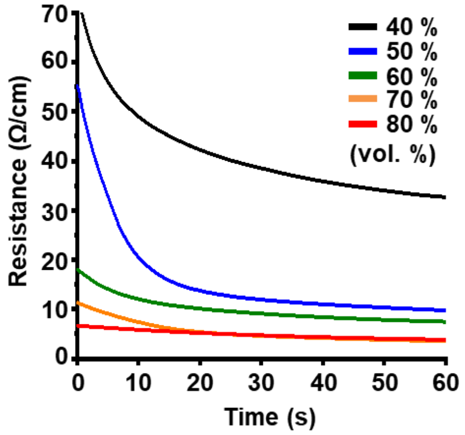

Supplementary Fig. 7 | Resistance variation as a function of the volume fraction of Ag after printing, without undergoing a sintering process.

The change in resistance over time for conductors with different volume fractions of Ag (40-80 %).

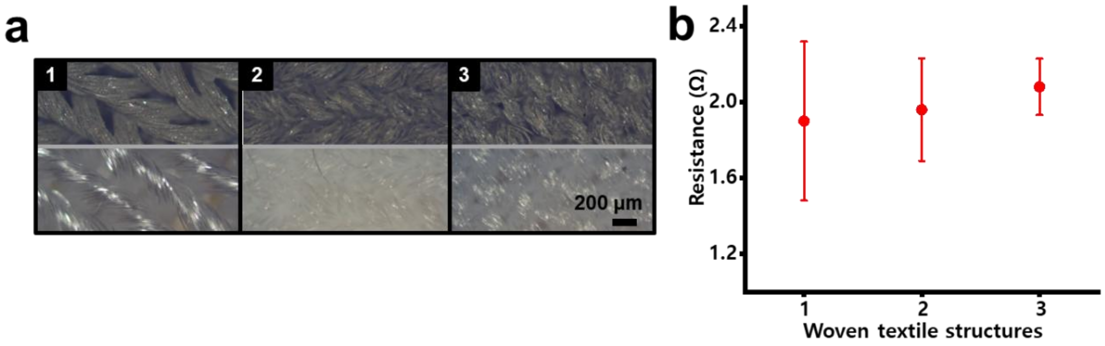

Supplementary Fig. 8 | Electrical performance of Ag-PU conductors on different textile substrates

a, Optical microscopy images of the textile substrate. The substrates were: Textile 1 (87% polyester, 13% spandex), Textile 2 (90% nylon, 10% polyurethane), and Textile 3 (88% polyester, 12% polyurethane). **b**, Absolute resistance of Ag-PU patterns (length 30 mm, pattern width 2 mm) printed on three textile substrates with varying compositions and weave/knit structures. The resulting mean resistances (1.90 Ω, 1.96 Ω, and 2.08 Ω, respectively) indicate that the conductor maintains consistent electrical performance across different substrate structures.

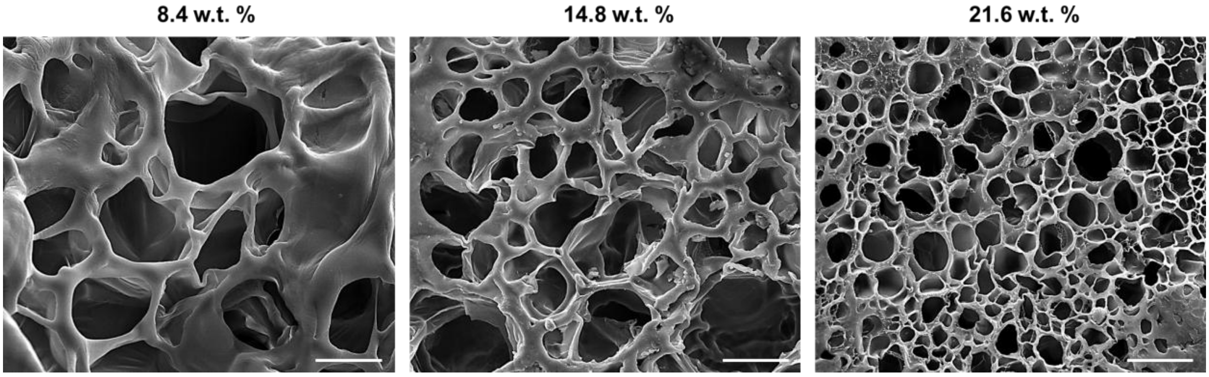

Supplementary Fig. 9 | SEM images of the DMCH

As the amount of PEDOT:PSS increased, the microporous structure was preserved, whereas the size of the small pores within the DMCH decreased. Scale bar, 20 μm.

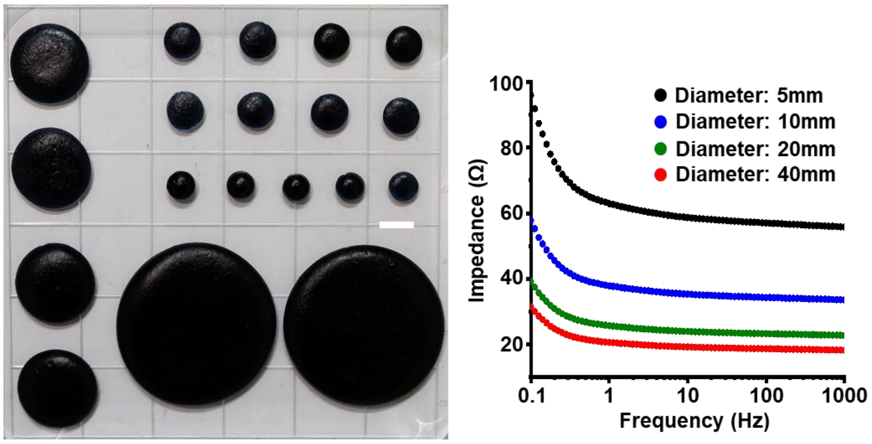

Supplementary Fig. 10 | Area dependency of impedance

Impedance changes according to the DMCH diameter (5, 10, 20, and 40 mm) This scalable process enables the fabrication of DMCHs with a wide range of array sizes and dimensions. Scale bar, 10 mm.

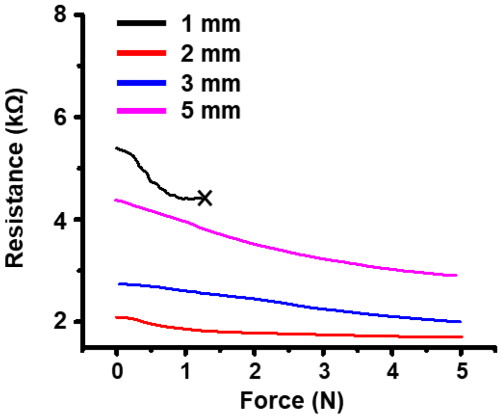

Supplementary Fig. 11 | Through-thickness (vertical) resistance as a function of applied compressive force for DMCH electrodes of varying thicknesses

Electrical characterization of the DMCH hydrogel electrode under a constant applied pressure of 5 N, where the through-thickness (vertical) resistance was measured using a universal testing machine (UTM).

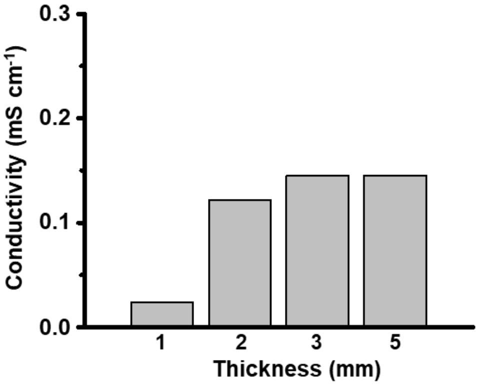

Supplementary Fig. 12 | Comparison of the conductivity of DMCH electrodes with different thicknesses.

Measured electrical conductivity of the DMCH hydrogel as a function of thickness.

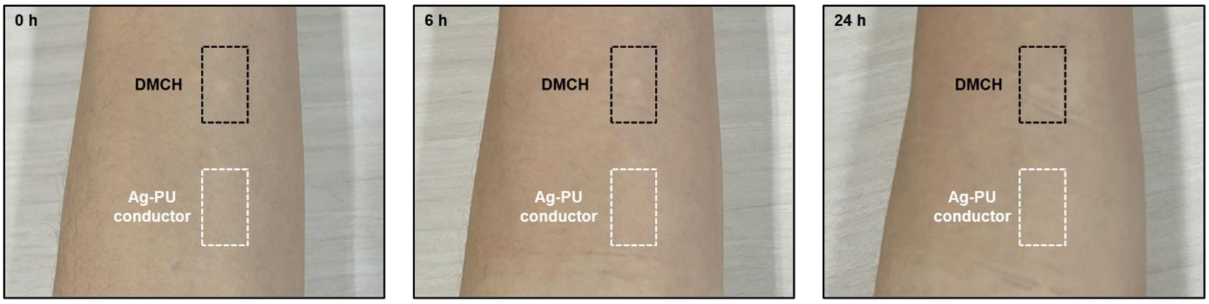

Supplementary Fig. 13 | 24-h skin-contact biocompatibility assessment of DMCH and Ag-PU conductor

Photographs of the skin after 24 h continuous contact with DMCH and the Ag-PU conductor.

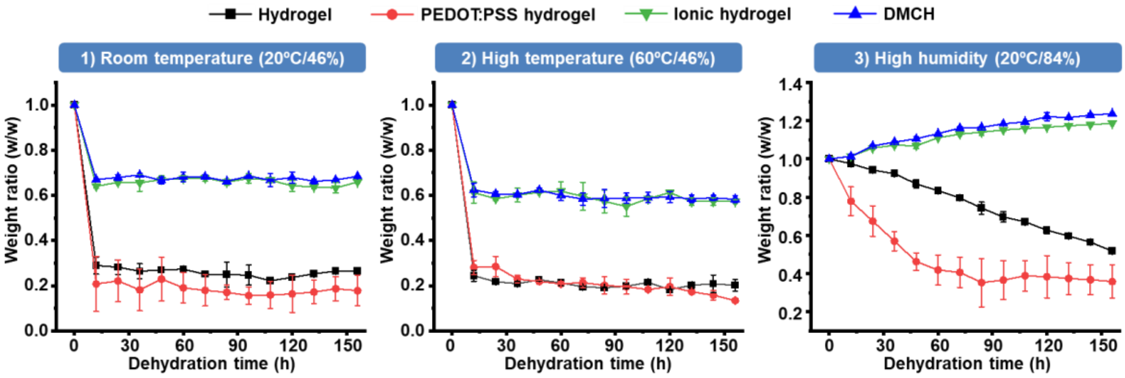

Supplementary Fig. 14 | Environmental stability of DMCH and reference hydrogels Time-dependent moisture content of four hydrogels (Hydrogel, PEDOT:PSS hydrogel, ionic hydrogel with LiCl, and DMCH) over 156 h under room-temperature, high-temperature, and high-humidity conditions.

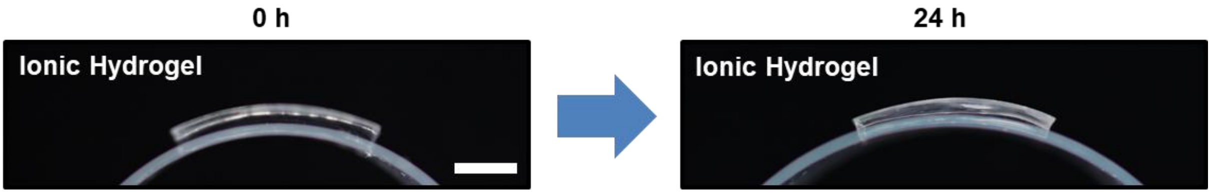

Supplementary Fig. 15 | Side view of the ionic hydrogel placed on a curved structure

Side view of the ionic hydrogel adhered to a surface with a curvature of 0.033 mm^{-1} at 0 h and 12 h under ambient indoor conditions of 20 °C and 44.2 % relative humidity. Scale bar, 8 mm.

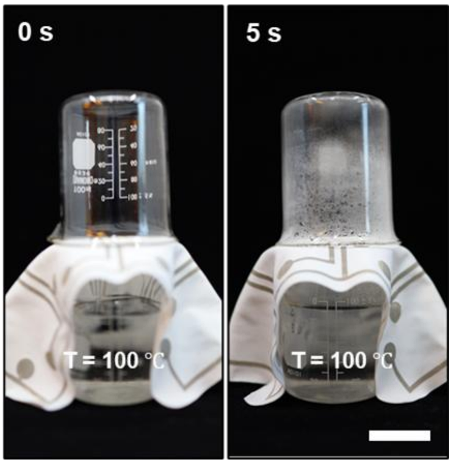

Supplementary Fig. 16 | Water vapor transmission in patterned textile

Photograph showing rapid water vapor transmission through an Ag-PU conductor printed on a textile with the pattern. Scale bar, 35 mm.

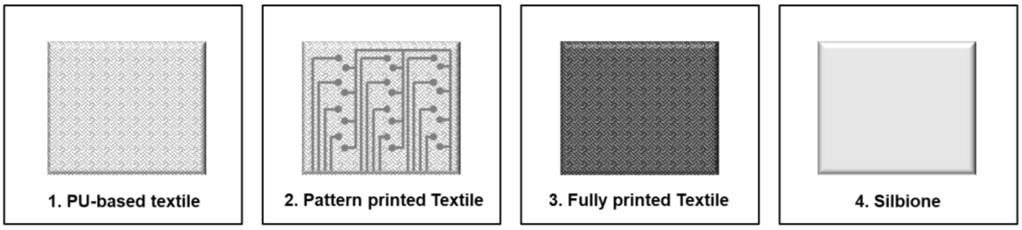

	1. PU textile	2. Pattern printed Textile	3. Fully printed Textile	4. Silbione
**100 Pa**	55.5	53.6	35.5	0
**300 Pa**	154	147	87.2	0
**600 Pa**	301	280	151	0
**900 Pa**	452	399	198	0

Air permeability (unit : $cm^3/cm^2/s$)

Supplementary Fig. 17 | Air permeability under various pressure conditions

Detailed air permeability measurements of the encapsulation materials were performed at pressures of 100, 300, 600, and 900 Pa.

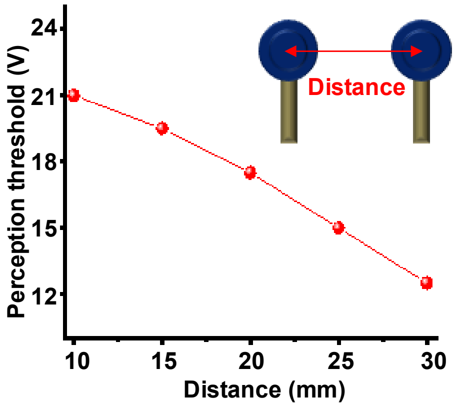

Supplementary Fig. 18 | The effect of inter-electrode distance on perception threshold

As the distance between the electrodes increased, the current flowed deeper into the skin, thereby lowering the perception threshold.

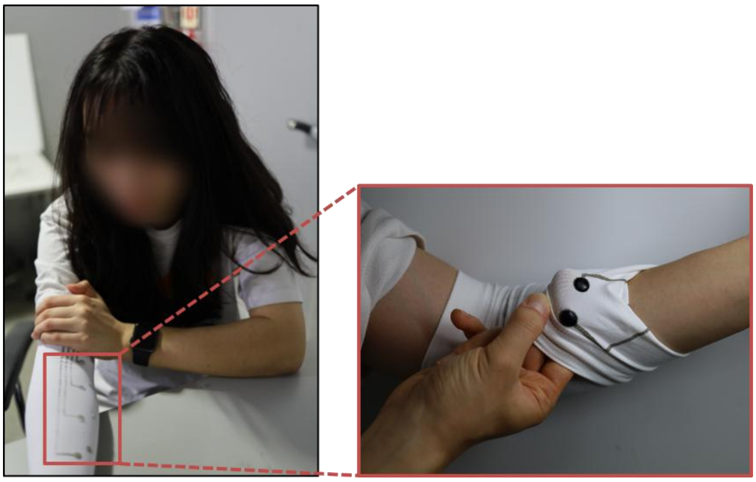

Supplementary Fig. 19 | Participant performing the sensory perception task

The participants underwent sensory perception tests using a textile-based electrical stimulation system.

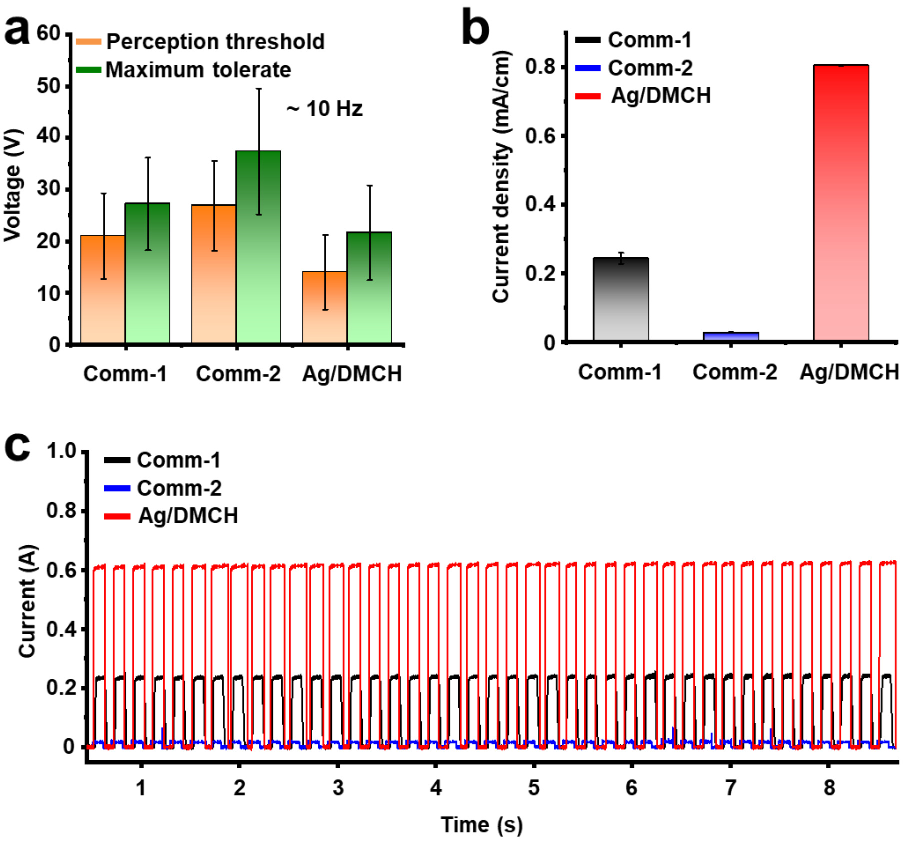

Supplementary Fig. 20 | Comparison with commercial electrodes

a, Comparison of the perception threshold voltage and maximum tolerable voltage of the Ag-PU conductor/DMCH and commercial electrodes. The Ag-PU conductor/DMCH sensed sensations at relatively low voltages. **b**, Current value per area flowing through the skin under the same voltage and frequency conditions. **c**, Current flowing through the skin under the same voltage and frequency conditions. The Ag-PU conductor/DMCH showed effective current efficiency at low voltages owing to its high conductivity.

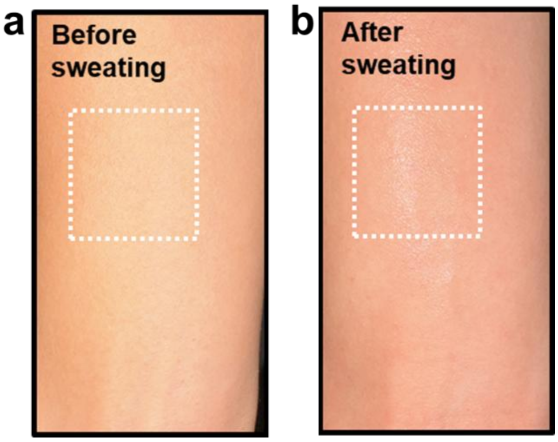

Supplementary Fig. 21 | Sweat accumulation after running

a, A photograph of the forearm before exercise. **b**, A photograph of the forearm after 10 min of running followed by 10 min of rest (13 °C, 66% relative humidity), showing visible sweat accumulation.

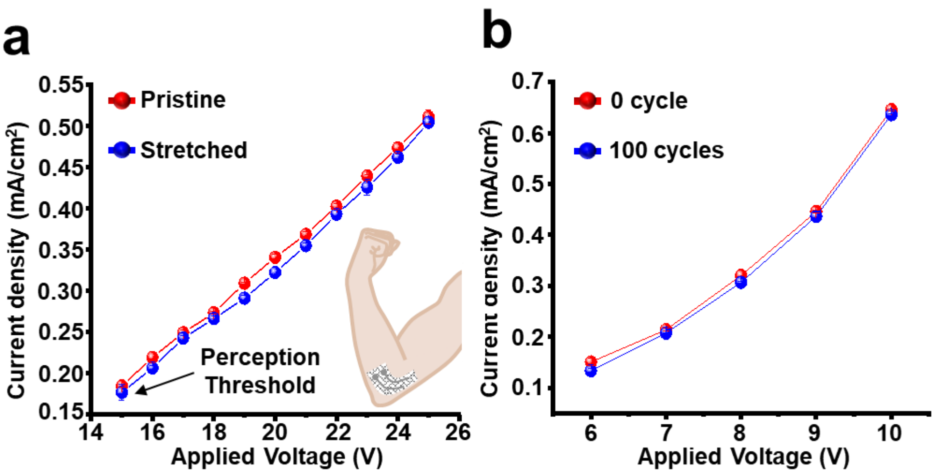

Supplementary Fig. 22 | Mechanical texting of the TESS

a, Comparison of current densities in the textile-based electrical stimulation system attached to the elbow before and after being stretched to 30 % strain. **b**, Comparison of the textile-based electrical stimulation system before and after 30 % 100-time cyclic stretching test ($n = 2$; point, mean; error bars, s.d.).

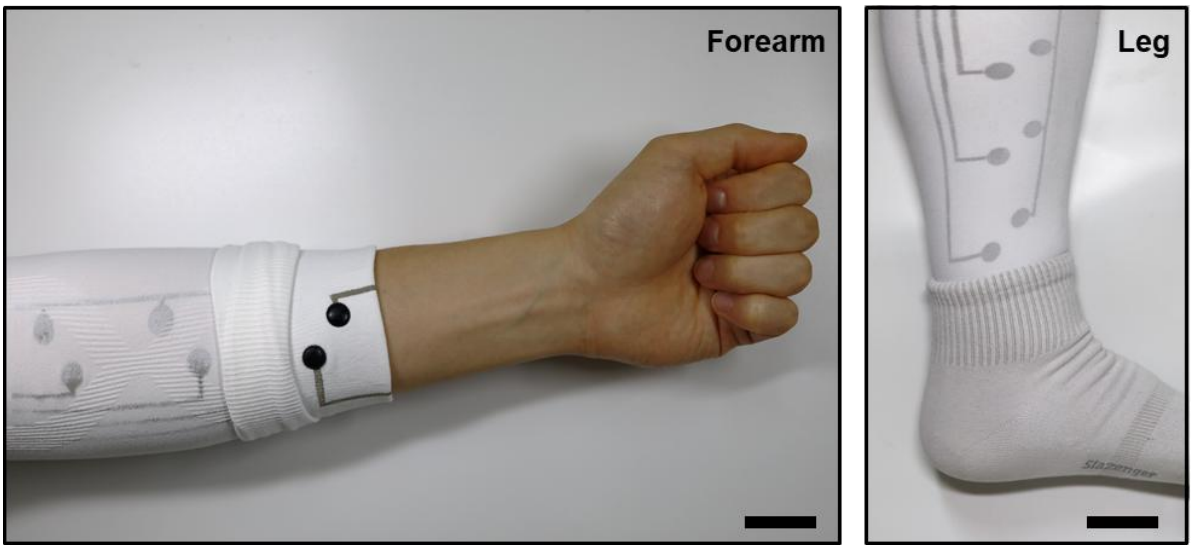

Supplementary Fig. 23 | Commercial arm sleeve and leg sleeve with a printed textile-based haptic system

Photograph of wearing 4 × 2 array and 4 × 1 array electrical stimulation systems manufactured on arm and leg cover sleeves, respectively. Scale bars, 20 mm.

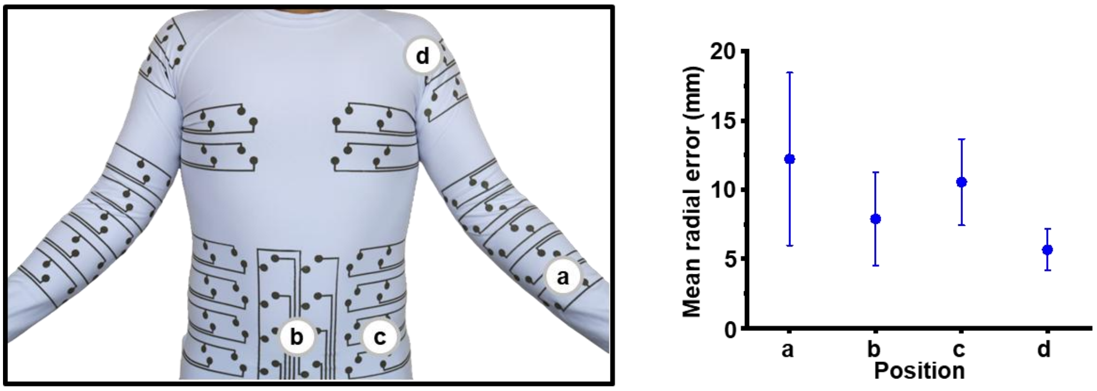

Supplementary Fig. 24 | Positional displacement of the TESS after repeated donning and doffing.

Average displacement of the electrode interface after 10 donning/doffing cycles across four major body regions.

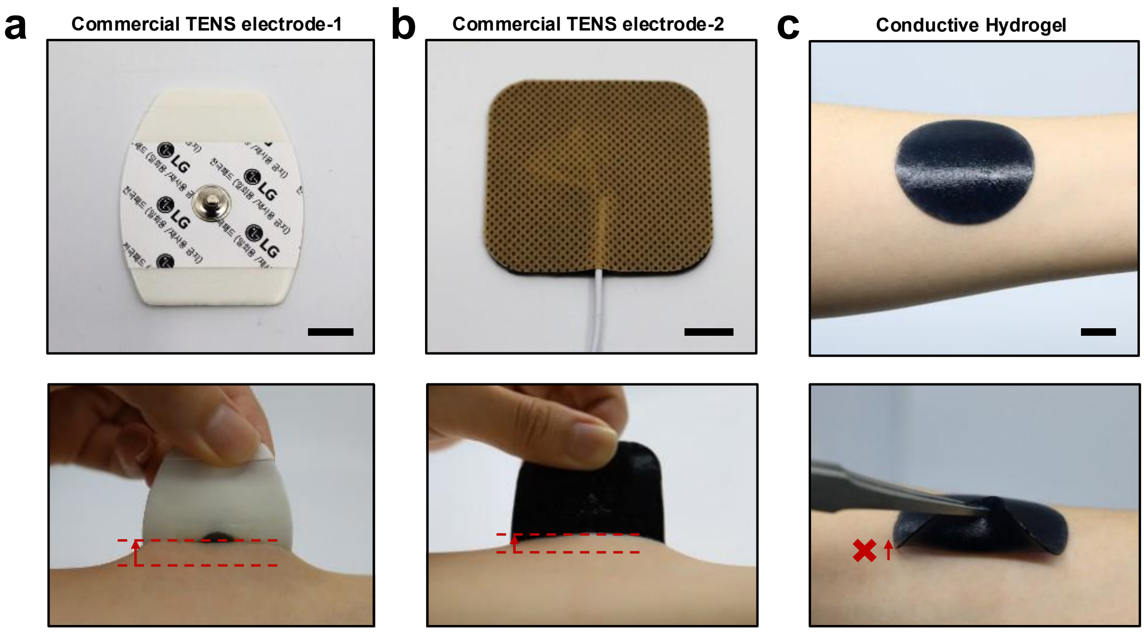

Supplementary Fig. 25 | Comparison of skin peel-off tests of electrodes

a-b, Strong adhesiveness causes the skin to stretch when the electrode is removed, irritating the skin. (**a**) Photograph showing the peel-off force of commercial electrode-1. The electrode is divided into a conductive part at the center and an adhesive part surrounding it. (**b**) Photograph showing the peel-off force of commercial electrode-2. This electrode had all combined conductive and adhesive layers. **c**, Photograph showing the peel-off force of DMCH. DMCH does not have strong adhesive properties or irritate the skin. The diameters of the electrodes used in the photographs were 40 mm. Scale bars, 10 mm.

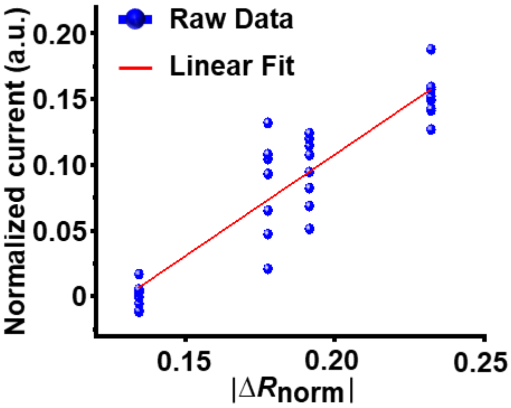

Supplementary Fig. 26 | Normalized additional currents induced by clothing pressure

Normalized additional current flows depending on the pressure, which induces variability in the haptic feedback intensity. A total of 8 repetitions were performed.

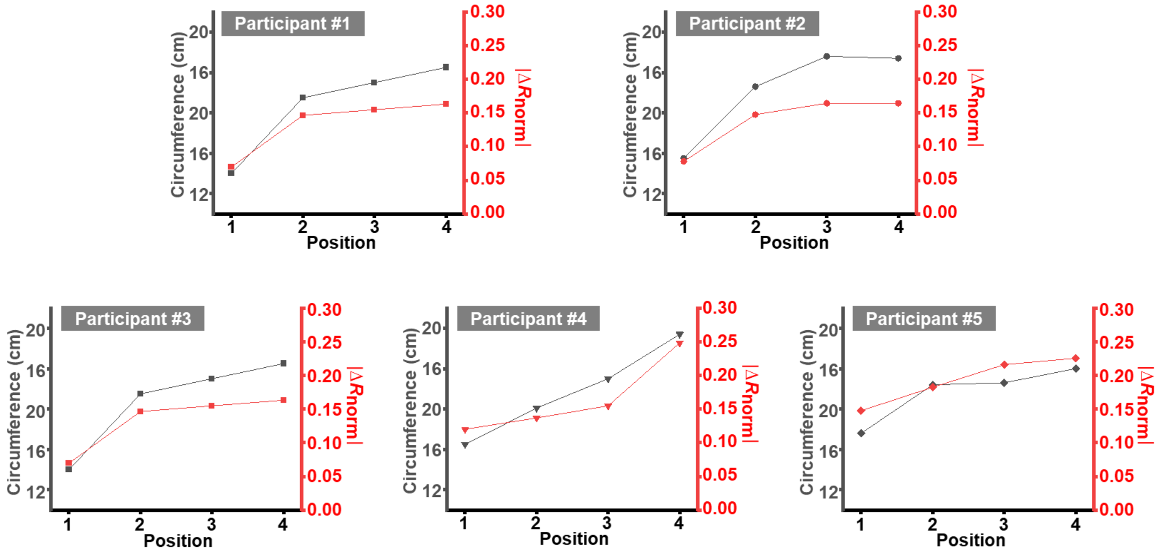

Supplementary Fig. 27 | Relationship between arm circumference and garment pressure sensing

Arm circumference and corresponding resistance changes of the pressure sensors at positions 1–4 along the arm for five participants. The arm circumference was measured using a flexible measuring tape.

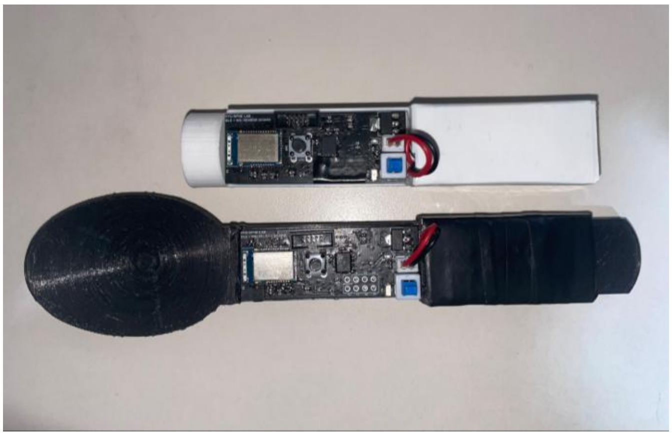

Supplementary Fig. 28 | Photograph of two controllers for tremor measurement

A black spoon-shaped controller was used for the tomato-moving task, whereas a white marker-type controller was employed for the spiral-drawing task.

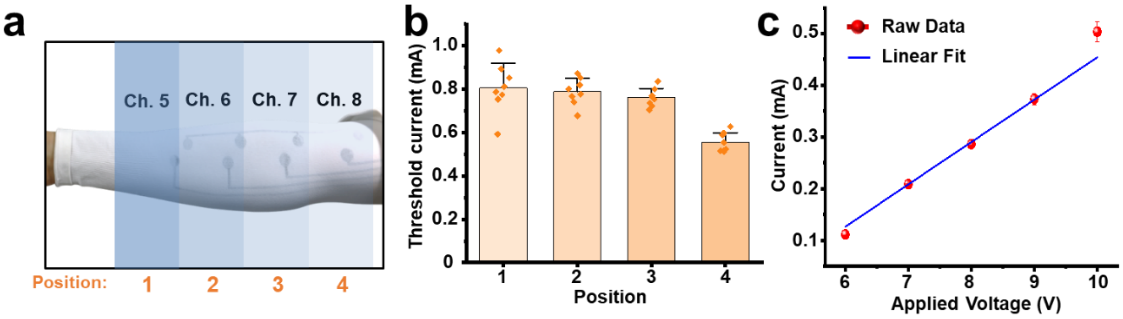

Supplementary Fig. 29 | Participant pre-electrical characteristics for haptic systems

a, Additional pressure induced current ($n = 8$). **b**, Electrical stimulation control system channel matching images for each arm position, along with the corresponding threshold current ($n = 8$; bar height, mean; error bars, s.d.). **c**, Linear current flowing through the skin as a function of input voltage.

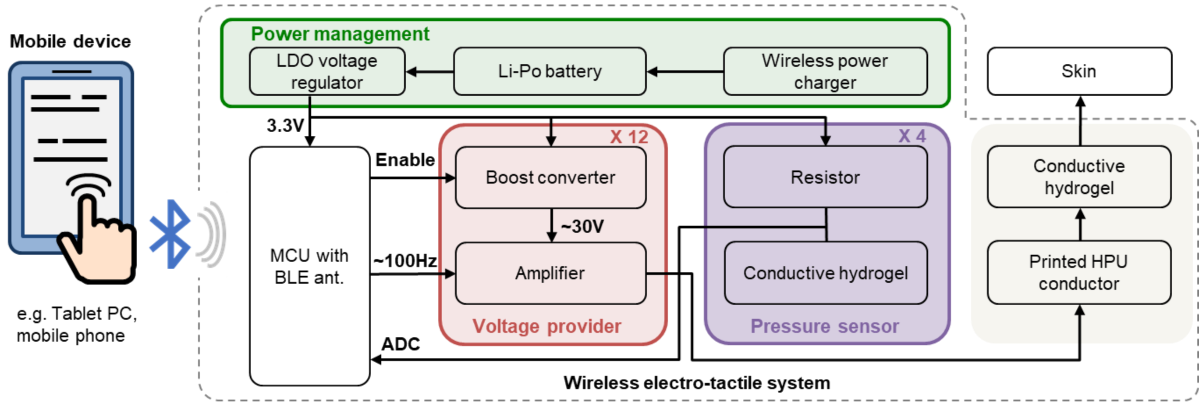

Supplementary Fig. 30 | Electrical stimulation control system

Block diagram and connection mechanisms of electrical stimulation system and its wireless interface to a mobile device via BLE.

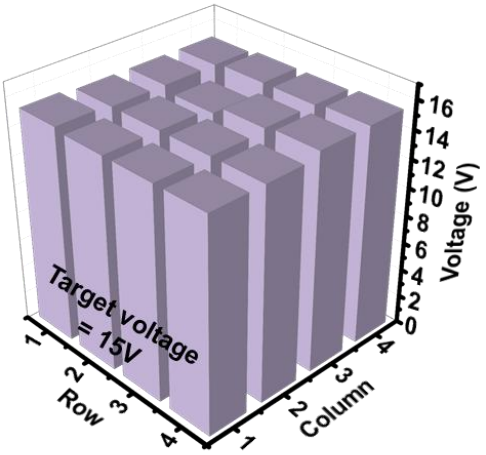

Supplementary Fig. 31 | Voltage uniformity in electrical stimulation circuits

Voltage uniformity in the electrical stimulation control system when targeting 15 V.

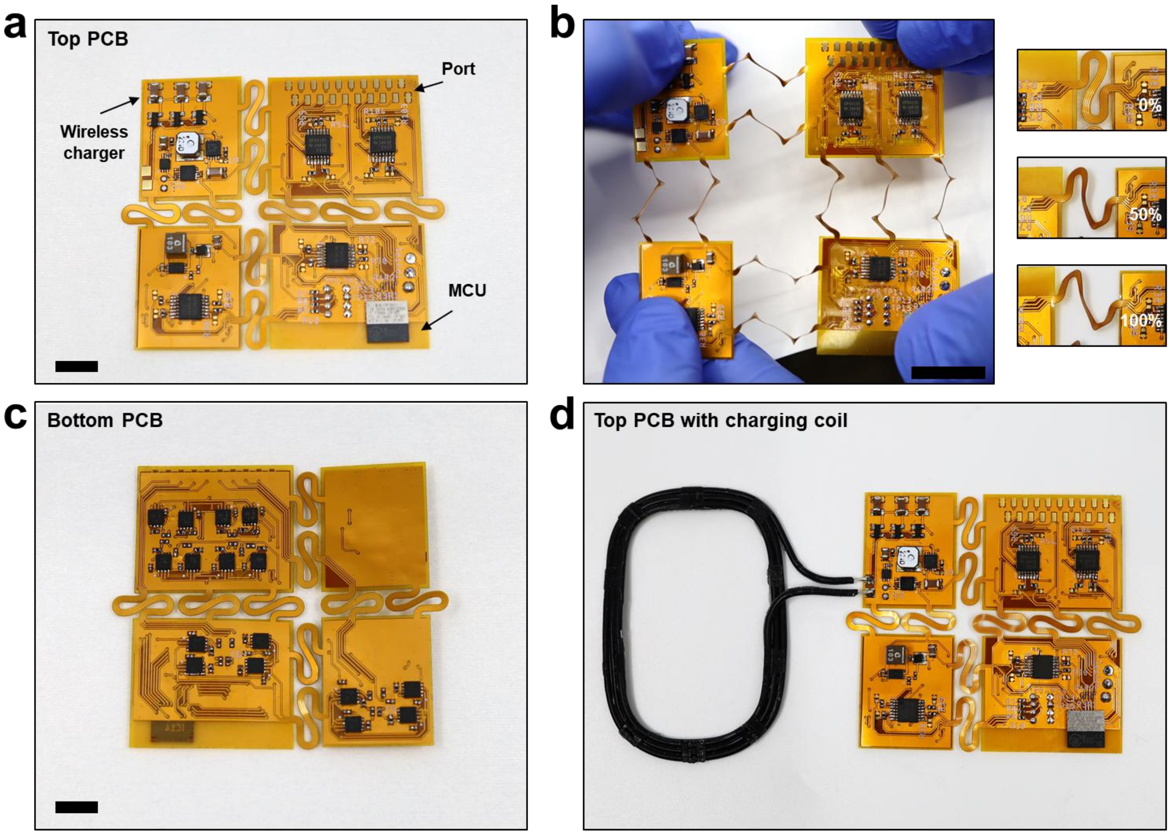

Supplementary Fig. 32 | Photograph of electro-tactile control circuit

a, Photograph of the electrical stimulation control circuit (top) separated into four squares. It consists of a microcontroller, wireless power charging, four voltage dividers for pressure sensing, and 12 channels. Scale bar, 20 mm. **b**, Photograph of electrical stimulation control circuit in stretched state. Serpentine electrical trace is stretched to 0 %, 50 %, and 100 % (right). Scale bar, 20 mm. **c**, Photograph of electrical stimulation control circuit (bottom). **d**, Photograph of electrical stimulation control circuit with charging coil.

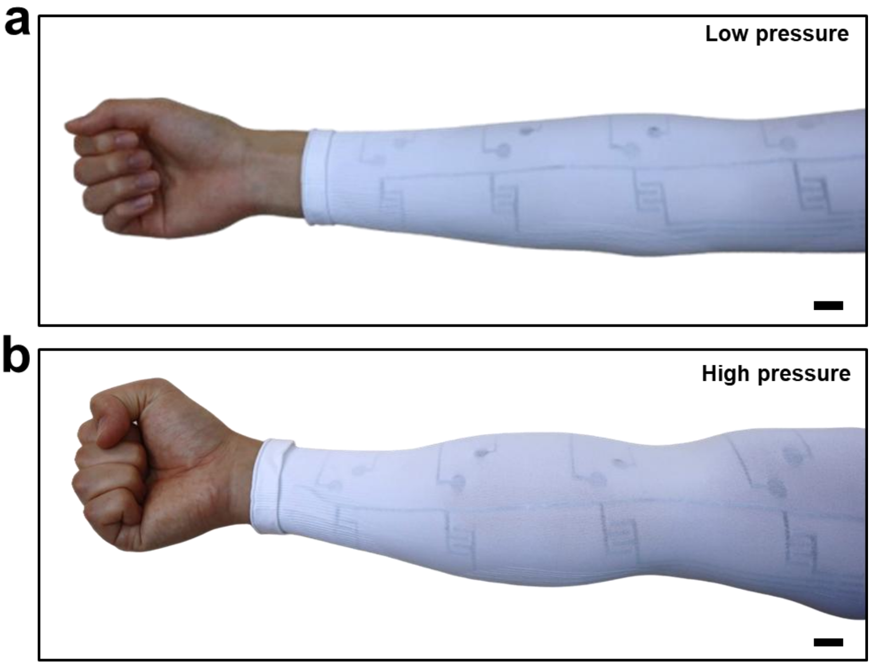

Supplementary Fig. 33 | Photograph of a participant wearing a forearm-based TESS system featuring an array of four electrotactile stimulator and pressure sensor pairs printed on a sleeve-type pressure garment

a, A photograph of the garment worn on a participant with a thinner arm, resulting in relatively low contact pressure measured by the sensors. **b**, A photograph of the same garment worn on a participant with a thicker arm, resulting in relatively high contact pressure. Scale bar, 20 mm.

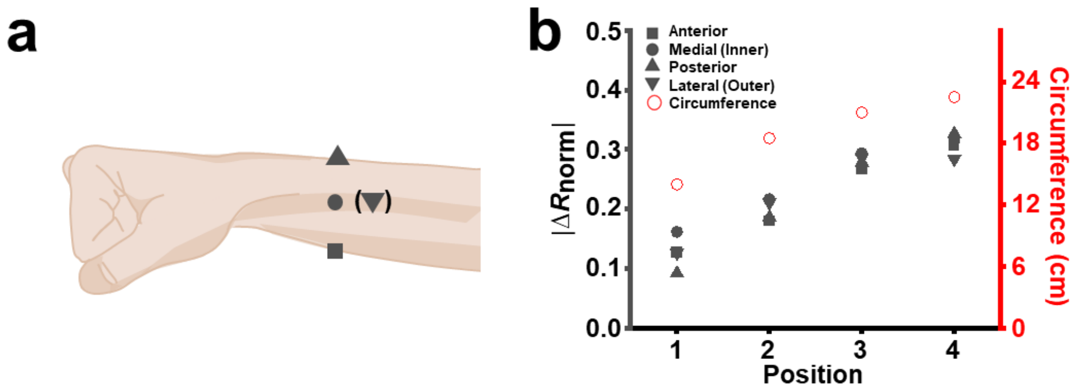

Supplementary Fig. 34 | Pressure variation analysis across the arm cross-section.

a, Pressure was measured at four circumferential locations (anterior, medial/inner, posterior, and lateral/outer) using the pressure sensor. **b**, Normalized resistance change (ΔRnorm) and arm circumference measured at four arm positions (1–4). The circumferential distances from the anterior location toward the inner direction to the remaining sensor locations were measured for each position (1: 0, 30, 70, 100 mm; 2: 0, 40, 95, 150 mm; 3: 0, 50, 100, 160 mm; 4: 0, 70, 120, 170 mm).

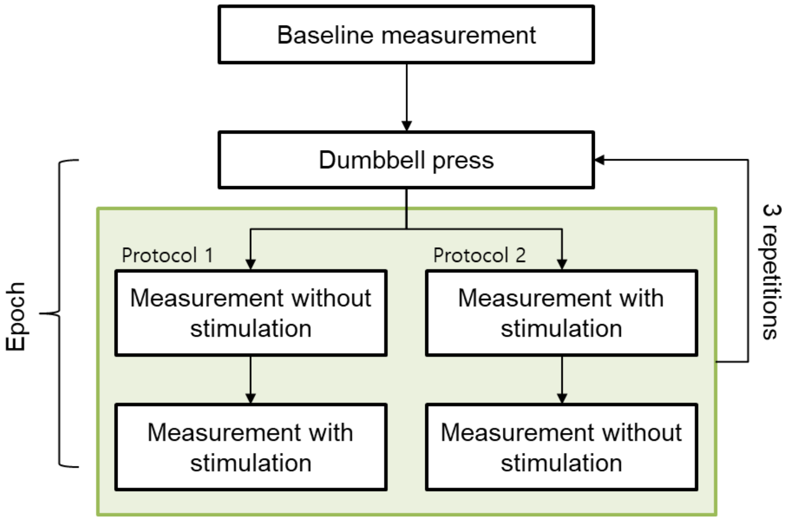

Supplementary Fig. 35 | Flowchart of the drawing an Archimedean spiral experiment

Initially, baseline measurements were obtained without electrical stimulation or tremor induction. This was followed by a series of dumbbell-based exercises designed to induce artificial tremors. Measurements were taken under both conditions, with and without electrical stimulation, when tremor was present. To minimize the influence of the measurement order, two protocols were randomly implemented: protocol 1, in which the condition without electrical stimulation was measured first, and protocol 2, in which the condition with electrical stimulation was measured first. After each measurement, the procedure was repeated three times.

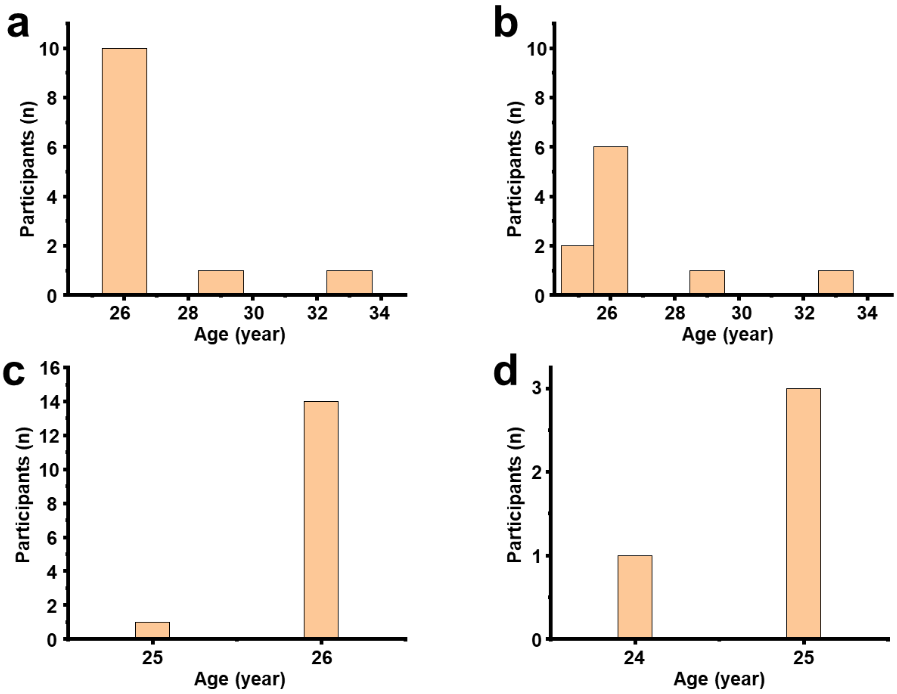

Supplementary Fig. 36 | Age distribution of participants in the user study

Histogram showing the number of participants as a function of age (years) for all user studies conducted in this work. **a**, Tomato-moving task. **b**, Spiral-drawing task. **c**, Human participant sensory perception test. **d**, Sweat test.

reference	Materials	Conductivity (S cm^{-1})	Heat treatment
[16]	Ag nanowire/ polydimethylsiloxane	56.82	100 ℃ for 2h
[17]	Ag flake/ vegetable oil-based polyurethane	12,833	60 ℃ for 3h
[18]	Ag nanowire/ thermoplastic polyurethane	5,114	120 ℃ for 30min
[19]	Ag nanowire/ thermoplastic polyurethane	3,668	80 ℃
[20]	Ag nanowire/ double-covered yarn (cotton fiber)	4,018	80 ℃ for 4h
[21]	Ag nanoparticle/ polydopamine	4,058	50 ℃ for a day
[22]	Poly(ethylene glycol) methyl ether appended to thioctic acid-modified AgNWs/ thermoplastic polyurethane	14,205	155 ℃ for 30min
[23]	Ag nanoparticle/ poly (styrene-block-butadiene-block-styrene)	5,400	No
[24]	Ag powder/ poly(vinylidene fluoride-co-hexafluoropropylene)	3,246	No
[24]	Ag flake/ poly(vinylidene fluoride-co-hexafluoropropylene)	3,116	No
This work	Ag flake/ thermoplastic polyurethane	5,411	No

Supplementary Table 1 | Conductivity comparison of Ag-based textile inks.

Reported conductivities of Ag-based inks on textile substrates, benchmarked against this work.

reference	Electrode type	Textile substrate	Electrode area
[25]	Silver/ carbon paste	Polyester 76 %, spandex 24 %	314 mm^2
[26]	Silver/ carbon paste	Polyester 88 %, spandex 12 %	314 mm^2
[27]	Screen-printed silver ink	Cotton knit/ polyester	600 mm^2
This work	Ag-PU conductor/ DMCH	Polyster 87 %, spandex 13 %	78.53 mm^2

Supplementary Table 2 | Comparison of reported textile-based electrodes (type, area, substrate)

Representative textile-based electrodes reported in the literature, including electrode type, electrode area, and substrate type.

a

**Equation**	y = a + b*x		
**Pearson's r**	0.9957		
**Adj. R-Square**	0.98857		
		**Value**	**Standard Error**
**Y**	**Intercept**	-3.6456E-4	3.11292E-5
	**Slope**	8.1874E-5	4.39537E-6

b

**Equation**	y = a + b*x		
**Pearson's r**	0.91154		
**Adj. R-Square**	0.82527		
		**Value**	**Standard Error**
**Y**	**Intercept**	-2E-4	2.37825E-5
	**Slope**	0.00154	1.26995E-4

Supplementary Table 3 | Detailed table of fitting equations for participant pre-electrical characteristics for haptic systems

a, Voltage conversion equation based on the participant current. **b**, Equation for deriving additional current induced by participant pressure.

Sensation	Detail
Touch	Light touch similar to tapping on a smartphone screen.
Tickling	Ticklish sensation, as if ants were crawling across the skin.
Roughness	Soft, textured feeling reminiscent of petting a cat or dog.
Pressure	Gentle, sustained pressure, akin to someone softly pressing with their hand.

Supplementary Table 4 | Detailed description of the four senses

Participants were asked to rate the following four sensations in detail: intensity and frequency.

reference	Feedback compensation	Stimuli point	Target	Stimulation coverage area	Device substrate	Haptic method	stretchability
[28]	x	21	Forearm	Medium	silicone rubber	electrotactile	High
[29]	x	9	Hand	Medium	Silicone	Pneumatic & vibrotactile	High
[30]	Current monitoring	25	Fingertip	Small	Flexible PCB	electrotactile	Medium
[31]	x	32	Hand	Medium	PDMS	electrotactile	Medium
[32]	x	6	Hand	Medium	Fabric	electrotactile	Medium
[33]	x	4	Arm	Small	Polyimide	electrotactile	Medium
[34]	x	7	Fingertip	Small	Silicone membra ne	electrostatic	High
[35]	x	25	Any part	Medium	PET & Al	electrostatic	Medium
[36]	Detecting pressure through the pressure-sensitive transistor	100	Fingertip	Small	Glass	electrotactile	Low
This work	Resistive pressure sensing	4	Full-body	Large	PU-based textile	electrotactile	High

Supplementary Table 5 | Comparison of TESS with previously reported haptic systems. Summary of representative haptic systems reported in the literature, including whether feedback compensation is implemented, the number and area of stimulation sites, the targeted body region (e.g., fingertip, hand), and the device substrate type and stretchability.

Supplementary References

1 Zhu, L. *et al.* Self-Adhesive Elastic Conductive Ink with High Permeability and Low Diffusivity for Direct Printing of Universal Textile Electronics. *ACS nano* **18,** 34750-34762 (2024).

2 Wang, P. *et al.* Well-defined in-textile photolithography towards permeable textile electronics. *Nature communications* **15,** 887 (2024).

3 Ding, C. *et al.* Durability study of thermal transfer printed textile electrodes for wearable electronic applications. *ACS Applied Materials & Interfaces* **14,** 29144-29155 (2022).

4 Kim, S. H. *et al.* Ultrastretchable conductor fabricated on skin-like hydrogel–elastomer hybrid substrates for skin electronics. *Advanced Materials* **30**, 1800109 (2018).

5 Jin, H. *et al.* Highly durable nanofiber-reinforced elastic conductors for skin-tight electronic textiles. *Acs Nano* **13**, 7905-7912 (2019).

6 Matsuhisa, N. *et al.* Printable elastic conductors with a high conductivity for electronic textile applications. *Nature communications* **6**, 7461 (2015).

7 Maeda, R. *et al.* The conducting fibrillar networks of a PEDOT: PSS hydrogel and an organogel prepared by the gel-film formation process. *Nanotechnology* **32**, 135403 (2021).

8 Greczynski, G., Kugler, T. & Salaneck, W. Characterization of the PEDOT-PSS system by means of X-ray and ultraviolet photoelectron spectroscopy. *Thin Solid Films* **354**, 129-135 (1999).

9 Andrei, V. *et al.* Size Dependence of Electrical Conductivity and Thermoelectric Enhancements in Spin-Coated PEDOT: PSS Single and Multiple Layers. *Advanced Electronic Materials* **3**, 1600473 (2017).

10 Nardes, A., Kemerink, M. & Janssen, R. Anisotropic hopping conduction in spin-coated PEDOT: PSS thin films. *Physical Review B—Condensed Matter and Materials Physics* **76**, 085208 (2007).

11 Carter, J. L., Kelly, C. A., Marshall, J. E. & Jenkins, M. J. Effect of thickness on the electrical properties of PEDOT: PSS/Tween 80 films. *Polymer Journal* **56**, 107-114 (2024).

12 Dijk, G., Ruigrok, H. J. & O'Connor, R. P. Influence of PEDOT: PSS coating thickness on the performance of stimulation electrodes. *Advanced Materials Interfaces* **7**, 2000675 (2020).

13 Wicaksono, I. *et al.* A tailored, electronic textile conformable suit for large-scale spatiotemporal physiological sensing in vivo. *npj Flexible Electronics* **4**, 1-13 (2020).

14 Zhao, L., Li, X., Yu, J., Li, C. & Li, G. Compression sleeves design based on Laplace laws. *Journal of Textile Engineering & Fashion Technology* **2**, 314-320 (2017).

15 Accot, J. & Zhai, S. in *Proceedings of the ACM SIGCHI Conference on Human factors in computing systems.* 295-302.

16 Zhu, C.-H., Li, L.-M., Wang, J.-H., Wu, Y.-P. & Liu, Y. Three-dimensional highly conductive silver nanowires sponges based on cotton-templated porous structures for stretchable conductors. *RSC Advances* **7,** 51-57 (2017).

17 Lv, J. *et al.* Printed sustainable elastomeric conductor for soft electronics. *Nature Communications* **14,** 7132 (2023).

18 Zhao, H. *et al.* Ultrastretchable and washable conductive microtextiles by coassembly of silver nanowires and elastomeric microfibers for epidermal human–machine interfaces. *ACS Materials Letters* **3,** 912-920 (2021).

19 Zhu, H.-W. *et al.* Printable elastic silver nanowire-based conductor for washable electronic textiles. *Nano Research* **13,** 2879-2884 (2020).

20 Cheng, Y., Wang, R., Sun, J. & Gao, L. Highly conductive and ultrastretchable electric circuits from covered yarns and silver nanowires. *ACS nano* **9,** 3887-3895 (2015).

21 Niu, B., Hua, T. & Xu, B. Robust deposition of silver nanoparticles on paper assisted by polydopamine for green and flexible electrodes. *ACS Sustainable Chemistry & Engineering* **8,** 12842-12851 (2020).

22 Lu, Y. *et al.* High-performance stretchable conductive composite fibers from surface-modified silver nanowires and thermoplastic polyurethane by wet spinning. *ACS applied materials & interfaces* **10,** 2093-2104 (2018).

23 Park, M. *et al.* Highly stretchable electric circuits from a composite material of silver nanoparticles and elastomeric fibres. *Nature nanotechnology* **7,** 803-809 (2012).

24 La, T. G. *et al.* Two-layered and stretchable e-textile patches for wearable healthcare electronics. *Advanced healthcare materials* **7,** 1801033 (2018).

25 Kim, S., Lee, S. & Jeong, W. EMG measurement with textile-based electrodes in different electrode sizes and clothing pressures for smart clothing design optimization. *Polymers* **12,** 2406 (2020).

26 Kim, H., Rho, S., Han, S., Lim, D. & Jeong, W. Fabrication of textile-based dry electrode and analysis of its surface EMG signal for applying smart wear. *Polymers* **14,** 3641 (2022).

27 Nigusse, A. B., Malengier, B., Mengistie, D. A., Tseghai, G. B. & Van Langenhove, L. Development of washable silver printed textile electrodes for long-term ECG monitoring. *Sensors* **20,** 6233 (2020).

28 Shi, Y. *et al.* Self-powered electro-tactile system for virtual tactile experiences. *Science Advances* **7,** eabe2943 (2021).

29 Liu, M. *et al.* Tactile sensing and rendering patch with dynamic and static sensing and haptic feedback for immersive communication. *ACS Applied Materials & Interfaces* **16,** 53207-53219 (2024).

30 Lin, W. *et al.* Super-resolution wearable electrotactile rendering system. *Science advances* **8**, eabp8738 (2022).

31 Yao, K. *et al.* Encoding of tactile information in hand via skin-integrated wireless haptic interface. *Nature Machine Intelligence* **4**, 893-903 (2022).

32 Xu, G. *et al.* Self-powered electrotactile textile haptic glove for enhanced human-machine interface. *Science advances* **11,** eadt0318 (2025).

33 Xu, B. *et al.* An epidermal stimulation and sensing platform for sensorimotor prosthetic control, management of lower back exertion, and electrical muscle activation. *Advanced Materials (Deerfield Beach, Fla.)* **28,** 4462 (2015).

34 Chen, S., Chen, Y., Yang, J., Han, T. & Yao, S. Skin-integrated stretchable actuators toward skin-compatible haptic feedback and closed-loop human-machine interactions. *npj Flexible Electronics* **7,** 1 (2023).

35 Leroy, E. & Shea, H. Hydraulically amplified electrostatic taxels (HAXELs) for full body haptics. *Advanced Materials Technologies* **8,** 2300242 (2023).

36 Lim, K. *et al.* Interference haptic stimulation and consistent quantitative tactility in transparent electrotactile screen with pressure-sensitive transistors. *Nature communications* **15,** 7147 (2024).
