# Supplementary material for: A lightweight durable full-body electrical stimulation suit for haptic feedback and therapeutic applications
Source: Nat Commun. 2026 Apr 2;17:4764. doi: 10.1038/s41467-026-71171-y (PMC13216600; doi:10.1038/s41467-026-71171-y)
Supplement: Supplementary file 2 — Reporting Summary [file 41467_2026_71171_MOESM2_ESM.pdf]

nature portfolio

Corresponding author(s): Yei Hwan Jung

Last updated by author(s): Jan 29, 2026

Reporting Summary

Nature Portfolio wishes to improve the reproducibility of the work that we publish. This form provides structure for consistency and transparency in reporting. For further information on Nature Portfolio policies, see our Editorial Policies and the Editorial Policy Checklist.

Statistics

For all statistical analyses, confirm that the following items are present in the figure legend, table legend, main text, or Methods section.

n/a	Confirmed	
[ ]	[x]	The exact sample size (*n*) for each experimental group/condition, given as a discrete number and unit of measurement
[ ]	[x]	A statement on whether measurements were taken from distinct samples or whether the same sample was measured repeatedly
[ ]	[x]	The statistical test(s) used AND whether they are one- or two-sided<br>*Only common tests should be described solely by name; describe more complex techniques in the Methods section.*
[x]	[ ]	A description of all covariates tested
[x]	[ ]	A description of any assumptions or corrections, such as tests of normality and adjustment for multiple comparisons
[ ]	[x]	A full description of the statistical parameters including central tendency (e.g. means) or other basic estimates (e.g. regression coefficient) AND variation (e.g. standard deviation) or associated estimates of uncertainty (e.g. confidence intervals)
[x]	[ ]	For null hypothesis testing, the test statistic (e.g. *F*, *t*, *r*) with confidence intervals, effect sizes, degrees of freedom and *P* value noted<br>*Give P values as exact values whenever suitable.*
[x]	[ ]	For Bayesian analysis, information on the choice of priors and Markov chain Monte Carlo settings
[x]	[ ]	For hierarchical and complex designs, identification of the appropriate level for tests and full reporting of outcomes
[x]	[ ]	Estimates of effect sizes (e.g. Cohen's *d*, Pearson's *r*), indicating how they were calculated

Our web collection on statistics for biologists contains articles on many of the points above.

Software and code

Policy information about availability of computer code

Data collection	DMM751, Keithley Instruments Software
Data analysis	SEGGER Embedded Studio (v5.44)

For manuscripts utilizing custom algorithms or software that are central to the research but not yet described in published literature, software must be made available to editors and reviewers. We strongly encourage code deposition in a community repository (e.g. GitHub). See the Nature Portfolio guidelines for submitting code & software for further information.

Data

Policy information about availability of data

All manuscripts must include a data availability statement. This statement should provide the following information, where applicable:

- Accession codes, unique identifiers, or web links for publicly available datasets
- A description of any restrictions on data availability
- For clinical datasets or third party data, please ensure that the statement adheres to our policy

The data supporting the findings of this study are included in the paper and its Supplementary Information or are available from the corresponding author upon reasonable request.

Research involving human participants, their data, or biological material

Policy information about studies with human participants or human data. See also policy information about sex, gender (identity/presentation), and sexual orientation and race, ethnicity and racism.

Reporting on sex and gender	Sex was recorded as a biological attribute, and participant sex is reported in the manuscript. The study was not designed or powered to assess sex-specific effects; therefore, analyses were not stratified by sex.
Reporting on race, ethnicity, or other socially relevant groupings	Race and ethnicity were not collected, and no analyses were conducted based on race/ethnicity.
Population characteristics	A total of 15 adults with ages ranging from 24-33 years have participated in this study.
Recruitment	Participants were recruited through Hanyang University advertisements and volunteered to participate. As recruitment was based on self-selection and convenience sampling, the sample may not be representative of the general population.
Ethics oversight	The experimental procedures involving human subjects were approved by the Hanyang University Institutional Review Board (HYUIRB-202511-004).

Note that full information on the approval of the study protocol must also be provided in the manuscript.

Field-specific reporting

Please select the one below that is the best fit for your research. If you are not sure, read the appropriate sections before making your selection.

[x] Life sciences [ ] Behavioural & social sciences [ ] Ecological, evolutionary & environmental sciences

For a reference copy of the document with all sections, see nature.com/documents/nr-reporting-summary-flat.pdf

Life sciences study design

All studies must disclose on these points even when the disclosure is negative.

Sample size	For each sensation test data were collected from each participant at least twice and up to four times to obtain meaningful assessment outcomes. Each experiment was repeated using a minimum of 1 and a maximum of 15 participants, with the sample sizes chosen based on prior studies. [Sci. Adv.10,eadq9575(2024).] [Nat Mach Intell 4, 893–903 (2022).]
Data exclusions	No data were excluded.
Replication	In this study, up to four biologically independent experiments were performed for each condition, and all results were reproduced reliably across up to four independent repeats.
Randomization	All samples were allocated randomly into experminetal groups.
Blinding	As blocking visual information is an important issue in tactile perception experiments, all experiments were blinded: no surgical or chemical intervention was used, but rather a physical barrier (blindfold, cloth, etc.) to block the view.

Reporting for specific materials, systems and methods

We require information from authors about some types of materials, experimental systems and methods used in many studies. Here, indicate whether each material, system or method listed is relevant to your study. If you are not sure if a list item applies to your research, read the appropriate section before selecting a response.

Materials & experimental systems

n/a	Involved in the study	
[x]	[ ]	Antibodies
[x]	[ ]	Eukaryotic cell lines
[x]	[ ]	Palaeontology and archaeology
[x]	[ ]	Animals and other organisms
[x]	[ ]	Clinical data
[x]	[ ]	Dual use research of concern
[x]	[ ]	Plants

Methods

n/a	Involved in the study	
[x]	[ ]	ChIP-seq
[x]	[ ]	Flow cytometry
[x]	[ ]	MRI-based neuroimaging

Plants

Seed stocks	The study did not involve plant samples
Novel plant genotypes	The study did not involve plant samples
Authentication	The study did not involve plant samples
